# *C. elegans* SWAN-1 Binds to EGL-9 and Regulates HIF-1-Mediated Resistance to the Bacterial Pathogen *Pseudomonas aeruginosa* PAO1

**DOI:** 10.1371/journal.ppat.1001075

**Published:** 2010-08-26

**Authors:** Zhiyong Shao, Yi Zhang, Qi Ye, Jenifer Neeta Saldanha, Jo Anne Powell-Coffman

**Affiliations:** Department of Genetics, Development, and Cell Biology, Iowa State University, Ames, Iowa, United States of America; Massachusetts General Hospital and Harvard Medical School, United States of America

## Abstract

*Pseudomonas aeruginosa* is a nearly ubiquitous human pathogen, and infections can be lethal to patients with impaired respiratory and immune systems. Prior studies have established that strong loss-of-function mutations in the *egl-9* gene protect the nematode *C. elegans* from *P. aeruginosa* PAO1 fast killing. EGL-9 inhibits the HIF-1 transcription factor via two pathways. First, EGL-9 is the enzyme that targets HIF-1 for oxygen-dependent degradation via the VHL-1 E3 ligase. Second, EGL-9 inhibits HIF-1-mediated gene expression through a VHL-1-independent mechanism. Here, we show that a loss-of-function mutation in *hif-1* suppresses *P. aeruginosa* PAO1 resistance in *egl-9* mutants. Importantly, we find stabilization of HIF-1 protein is not sufficient to protect *C. elegans* from *P. aeruginosa* PAO1 fast killing. However, mutations that inhibit both EGL-9 pathways result in higher levels of HIF-1 activity and confer resistance to the pathogen. Using forward genetic screens, we identify additional mutations that confer resistance to *P. aeruginosa*. In genetic backgrounds that stabilize *C. elegans* HIF-1 protein, loss-of-function mutations in *swan-1* increase the expression of hypoxia response genes and protect *C. elegans* from *P. aeruginosa* fast killing. SWAN-1 is an evolutionarily conserved WD-repeat protein belonging to the AN11 family. Yeast two-hybrid and co-immunoprecipitation assays show that EGL-9 forms a complex with SWAN-1. Additionally, we present genetic evidence that the DYRK kinase MBK-1 acts downstream of SWAN-1 to promote HIF-1-mediated transcription and to increase resistance to *P. aeruginosa*. These data support a model in which SWAN-1, MBK-1 and EGL-9 regulate HIF-1 transcriptional activity and modulate resistance to *P. aeruginosa* PAO1 fast killing.

## Author Summary

*Pseudomonas aeruginosa* is a common bacterial pathogen that can infect a wide range of animals. In some conditions, *P. aeruginosa* produces cyanide, a toxin that limits cellular capacity to metabolize oxygen and produce energy. The nematode *Caenorhabditis elegans* is a powerful genetic model system for understanding the mechanisms of stress response and pathogen resistance. Here, we show that HIF-1, a DNA-binding transcription factor that mediates cellular responses to low oxygen, can protect *C. elegans* from *P. aeruginosa* fast killing. Additionally, we identify *swan-1* as a gene that functions to inhibit HIF-1 activity and suppress *P. aeruginosa* resistance. The SWAN-1 protein binds directly to the oxygen-sensing EGL-9 enzyme that controls HIF-1 stability and activity. This study advances understanding of HIF-1 regulatory networks, defines connections between hypoxia response and *P. aeruginosa* fast killing, and provides new insights into mechanisms by which animals can resist this bacterial pathogen.

## Introduction

*Pseudomonas aeruginosa* is a ubiquitous bacterial pathogen that can infect a wide range of animals and plants, and hospital-acquired *P. aeruginosa* infections are often lethal to patients with respiratory ailments or immune system dysfunction [1], [2]. The cyanide produced by *P. aeruginosa* is thought to contribute to the potentially devastating effects of *P. aeruginosa* respiratory infections in cystic fibrosis patients [3]. Antibiotic-resistant strains of *P. aeruginosa* are becoming more prevalent, and it is increasingly important to understand the pathogenicity of this microbe and the mechanisms that enable resistance [4], [5].

During infection and inflammation, multicellular tissues must adapt to changing levels of oxygen. The hypoxia-inducible factor (HIF) transcription complex mediates most of the transcriptional responses to hypoxia (low oxygen) [6], [7]. While HIF transcription complexes have been shown to play key roles in mammalian innate immunity, the mechanisms by which HIF regulatory networks influence pathogenicity and disease progression are not yet fully understood [8], [9], [10], [11], [12].

In recent years, the nematode *Caenorhabditis elegans* has emerged as a powerful genetic system to study innate immunity and resistance to bacterial pathogens [13], [14], [15], [16], [17], [18]. Many of the genes that contribute to *C. elegans* pathogen resistance are evolutionarily conserved [19], [20], [21]. Interestingly, there is a strong correlation between *C. elegans* genes that mediate resistance to bacterial pathogens and genes that protect *C. elegans* from stresses and extend lifespan [22], [23], [24], [25], [26].

Loss-of-function mutations in the *C. elegans egl-9* gene enable the animals to survive fast killing by *P. aeruginosa* PAO1 [27], [28]. While some *Pseudomonas* strains (such as PA14 on NGM growth media) kill *C. elegans* slowly through colonization in the gut, logarithmically growing *P. aeruginosa* PAO1 emits cyanide and kills *C. elegans* within hours [14], [27], [28], [29]. *C. elegans egl-9* mutants are also resistant to Crystal or *Vibrio cholerae* pore-forming toxins [30]. The *egl-9* gene encodes a 2-oxoglutarate-dependent dioxygenase that hydroxylates the HIF-1 transcription factor. Once HIF-1 is hydroxylated, it interacts with the VHL-1 E3 ligase and is targeted for proteasomal degradation [31]. EGL-9 has also been shown to inhibit HIF-1 transcriptional activity via a *vhl-1*-independent pathway that has little or no requirement for EGL-9 hydroxylase activity [32], [33]. Moderate over-expression of HIF-1 has been shown to increase resistance to heat and to increase adult longevity in *C. elegans* [34], [35], [36], [37].

In this study, we directly test the hypothesis that increased expression and activation of the HIF-1 transcription factor in *egl-9* mutants protect *C. elegans* from *P. aeruginosa* PAO1 fast killing. We show that resistance to *P. aeruginosa* fast killing requires both stabilization of HIF-1 protein and derepression of HIF-1-mediated gene expression. Using forward genetic screens, we identify additional mutations that confer resistance to *P. aeruginosa* PAO1 fast killing. This leads to the discovery that SWAN-1 inhibits HIF-1 transcriptional activity and modulates resistance to *P. earuginosa* PAO1 fast killing. SWAN-1 is an evolutionarily conserved protein with WD40 repeats [38]. Further, we demonstrate that SWAN-1 interacts with EGL-9 protein in yeast two-hybrid and co-immunoprecipitation studies.

## Results

### *hif-1* is required for the *egl-9*-mediated resistance to PAO1 fast killing

**Figure 1:**
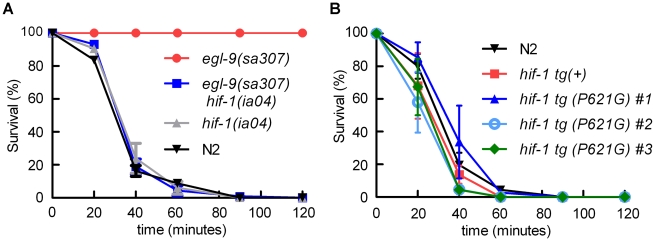
*egl-9*-mediated resistance to *Pseudomonas aeruginosa* PAO1 fast killing is mediated by *hif-1*. **A.** Percent *C. elegans* survival over time on *P. aeruginosa* PAO1. Wild-type N2 and *hif-1* mutants exhibit similar sensitivity to fast killing (>50% dead by 40 minutes), but 100% of animals carrying a strong loss-of-function mutation in *egl-9* survived after 2 hours. The *P. aeruginosa* resistance phenotype conferred by *egl-9(sa307)* was suppressed by the *hif-1(ia04)* strong loss-of-function allele. **B.** Stabilization of HIF-1 protein was not sufficient to confer resistance to *P. aeruginosa* PAO1 fast killing. The survival curves for strains expressing epitope-tagged HIF-1 or stabilized HIF-1(P621G) were similar to that of the wild-type strain. Three independently generated transgenic strains expressing HIF-1(P621G) were assayed. Each experiment was repeated at least three times with 30–50 animals for each strain. The error bars represent standard errors. These data are also presented in tabular format in Text S1.

The *egl-9(sa307)* strong loss-of-function mutation has been shown to protect *C. elegans* from *P. aeruginosa* PAO1 fast killing [27], [28] As shown in Figure 1A, wild-type animals are paralyzed when placed on *P. aeruginosa* PAO1, while *egl-9* mutant animals remain motile for several hours. We tested the hypothesis that *egl-9*-mediated resistance to fast killing required *hif-1* function. As shown in Figure 1A, the *hif-1(ia04)* loss-of-function allele totally suppressed the *egl-9*-mediated resistance phenotype. The rate at which the *egl-9, hif-1* double mutant was killed by the pathogen was very similar to the killing curves for wild-type or *hif-1(ia04)* animals (Figure 1A, Text S1). A prior study had shown that the fast killing of *C. elegans* by *P. aeruginosa* required cyanide synthesis [27]. Consistent with this, we found that while *P. aeruginosa* PAO1 killed wild-type or *hif-1*-deficient *C. elegans* within 2 hours, the hydrogen cyanide synthase mutant *P. aeruginosa* MP507 did not kill *C. elegans* in this time interval (Text S1).

We next investigated which EGL-9 functions were most critical to the *P. aeruginosa* PAO1 fast killing phenotype. EGL-9 regulates HIF-1 via at least two pathways: EGL-9 is the oxygen-sensitive enzyme that targets HIF-1 protein for degradation through the VHL-1 pathway, and EGL-9 inhibits HIF-1-mediated transcriptional activity by a *vhl-1*-independent mechanism [32], [33], [39]. We first tested the hypothesis that stabilization of HIF-1 protein was sufficient to increase resistance to *P. aeruginosa* PAO1 fast killing. The HIF-1(P621G) mutation precludes hydroxylation of HIF-1 by EGL-9 and stabilizes HIF-1 protein [31], [32], [34]. We assayed four transgenic strains, each expressing either wild-type HIF-1 or the HIF-1(P621G) stabilized protein. Remarkably, none of the transgenic strains were resistant to *P. aeruginosa* PAO1, as they died at rates similar to wild-type animals (Figure 1B, Text S1). Consistent with this result, the *vhl-1(ok161)* mutation did not protect *C. elegans* from fast killing (Text S1).

### Genetic screens identify mutations in *swan-1* and *rhy-1* that increase HIF-1-mediated transcription and protect *C. elegans* from *P. aeruginosa* PAO1 fast killing

The results thus far suggested that resistance to fast killing required multiple EGL-9 functions. To gain insight to the mechanisms by which EGL-9 repressed HIF-1 transcriptional activity and to better understand *P. aeruginosa* PAO1 pathogenicity, we conducted forward genetic screens. Using chemical or transposon-mediated mutagenesis, we screened for mutations in *C. elegans* that caused dramatic over-expression of HIF-1 target genes. As a primary screen, we assayed for the increased expression of P*nhr-57::*GFP, a reporter that is expressed at very low levels in wild-type animals and is expressed at high levels in *egl-9* mutants [32], [33]. These screens identified novel loss-of-function mutations in *rhy-1* (Text S1). Prior studies had shown that *rhy-1* encoded a multipass transmembrane protein, and loss-of-function mutations in *rhy-1* had been shown to elevate *hif-1* mRNA levels slightly and to increase HIF-1 transcriptional activity [33]. As shown in Table 1, animals that lacked *rhy-1* function were resistant to *P. aeruginosa* PAO1 fast killing, and *rhy-1*-mediated resistance was completely suppressed by the *hif-1(ia04)* strong loss-of-function mutation.

**Table 1:** Loss-of-function mutations in *egl-9* or *rhy-1* confer resistance to *P. aeruginosa* PAO1 fast killing.

Strains	Survival rate after 4 hours	Total animals	Experimental replicates
N2	3±1%	178	5
*rhy-1(ok1402)*	93±6%	178	5
*rhy-1(ok1402) hif-1(ia04)*	0±0%	142	4
*egl-9(sa307)*	100±0%	177	3
*egl-9(ia60)*	100±0%	91	4
*egl-9(ia58)*	100±0%	67	2
*rhy-1(ia59)*	100±0%	123	3
*rhy-1(ia63)*	99±1%	143	3
*rhy-1(ia64)*	100±0%	112	2
*rhy-1(ia62)*	96±1%	71	2

**Figure 2:**
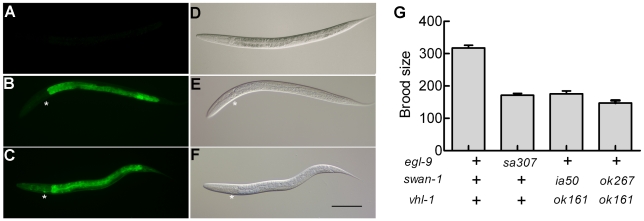
Phenotypic characterization of the *ia50* mutation. The *ia50* allele was isolated in a genetic screen for mutations that increased expression of P*nhr-57*:*:*GFP in animals expressing stabilized HIF-1. **A – F.** Fluorescent images are shown in A, B, C, and the corresponding DIC images are in D, E, F. **A, D.** Wild-type animals express the reporter at very low levels. **B, E.** In animals carrying the *vhl-1(ok161)* strong loss-of-function mutation, HIF-1 protein is stable, and the reporter is expressed throughout the intestine. **C, F.** *ia50 vhl-1(ok161)* double mutants express the reporter at higher levels and in more tissues, including the excretory cell and the hypodermis. Asterisks mark the position of the excretory cell, near the posterior bulb of the pharynx in the head. The scale bar represents 0.1 mm. **G.** Total brood sizes (numbers of progeny) were lower in *egl-9*-deficient animals and in double mutants carrying loss-of-function mutations in both *vhl-1* and *swan-1,* compared to wild-type *C. elegans*.

Reasoning that the effects of some mutations that derepressed HIF-1 activity might only be evident if HIF-1 protein were stable, we crossed a *vhl-1* loss-of-function mutation into the parental strain and screened for mutations that increased expression of the reporter. One such screen identified *ia50*, a mutation that enhanced expression of the P*nhr-57*::GFP reporter in *vhl-1(ok161)* mutants (compare Figure 2C to 2B). While *vhl-1(ok161)* animals over-expressed the reporter in the intestine, the *ia50* mutation expanded expression of P*nhr-57*::GFP to other tissues, including the hypodermis and the excretory cell (Figure 2C). The enhanced GFP expression phenotype of *ia50* was completely recessive. The *ia50, vhl-1(ok161)* double mutants had morphological defects that were similar to those seen in *egl-9* loss-of-function mutants, including egg-laying defects (data not shown). Additionally, both strains exhibited reduced fertility (Figure 2G).

**Figure 3:**
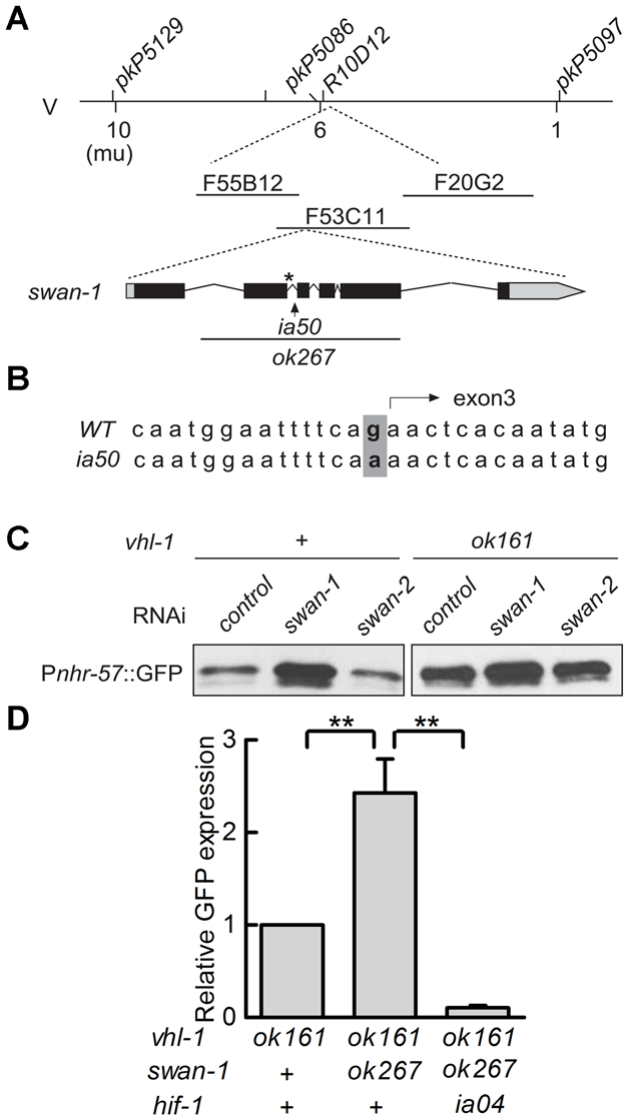
*ia50* is a loss of function mutation in the *swan-1* gene. **A.** Genetic mapping of the *ia50* mutation. Three-point mapping with the indicated single nucleotide polymorphisms placed *ia50* at +5.67 mu on chromosome V. Injection of a pool of 3 cosmids in the region (F20G2, F53C11, and F55B12) rescued the *ia50* homozygous mutants, as assayed by P*nhr-57*::GFP expression. This region includes the *swan-1* gene. *swan-1(ok267)* is a deletion mutation. **B.** *ia50* mutants carry a point mutation at the 3′ end of intron 2 of *swan-1*. This results in failure to splice out intron 2 from the mRNA and introduces an early stop codon, illustrated by an asterisk. **C.** Depletion of *swan-1* by bacterially-mediated RNAi increased expression of the P*nhr-57*::GFP reporter, as assayed by protein blots. In control experiments, RNAi for the related *swan-2* gene did not alter P*nhr-57*::GFP levels. **D.** The *swan-1(ok267)* mutation increased expression of the P*nhr-57*::GFP reporter in *vhl-1(ok161)* animals, and this phenotype was suppressed by the *hif-1(ia04)* strong loss-of-function mutation. P*nhr-57*::GFP levels were assayed by protein blots, and the error bars represent standard errors. **: P<0.01.

Genetic mapping with single-nucleotide polymorphisms placed *ia50* near +5.67 map units on chromosome five of the *C. elegans* genome (Figure 3A). Cosmid rescue experiments further delimited a genomic region that could restore a wild-type P*nhr-57*::GFP expression pattern to *ia50* mutant animals (Figure 3A). We sequenced the genes in this region and found that *ia50* mutants carried a single nucleotide mutation in the splice acceptor site for *swan-1* (F53C11.8) exon 3 (Figure 3A, 3B). Full-length cDNA sequencing confirmed that *ia50* mutants did not splice intron 2 from the *swan-1* mRNA, and this introduced an early stop codon. The gene name *swan-1* means “seven WD repeats, AN11 family”, and this family of genes includes Petunia AN11, Arabidopsis TTG1, zebrafish Wdr68, and human HAN11 [40], [41], [42], [43]. WD repeat proteins have beta propeller tertiary structures and often serve as platforms for the assembly of larger protein complexes. The *swan-1(ia50)* mutant allele is predicted to encode a truncated protein including only 2 of the WD repeats, and this suggests that it is a loss-of-function mutation.

To further test the hypothesis that the *ia50* mutant phenotype was due to defects in the *swan-1* gene, we used bacterially mediated RNAi to deplete *swan-1* mRNA. *swan-1* RNAi increased the expression of P*nhr-57*::GFP, as assayed by protein blots. In control experiments, RNAi for a neighboring gene, *swan-2*, did not change the expression of the reporter (compare to the empty vector control in Figure 3C).

Prior studies had characterized the *swan-1(ok267)* deletion mutation as a strong loss-of-function allele [38] (illustrated in Figure 3A). When HIF-1 protein was stabilized by the *vhl-1(ok161)* mutation, the *swan-1(ok267)* allele increased expression of the P*nhr-57*::GFP reporter (Figure 3D). This phenotype was suppressed by a loss-of-function mutation in *hif-1*. The *swan-1* deletion allele also reduced fertility in a *vhl-1* mutant background (Figure 2G). These similarities between the *swan-1(ia50)* and *swan-1(ok267)* phenotypes provided additional support for the conclusion that *ia50* was a loss-of-function mutation in the *swan-1* gene. For more in-depth analyses of *swan-1* function, we used the *swan-1(ok267)* deletion allele, as it had been characterized in prior studies [38].

**Figure 4:**
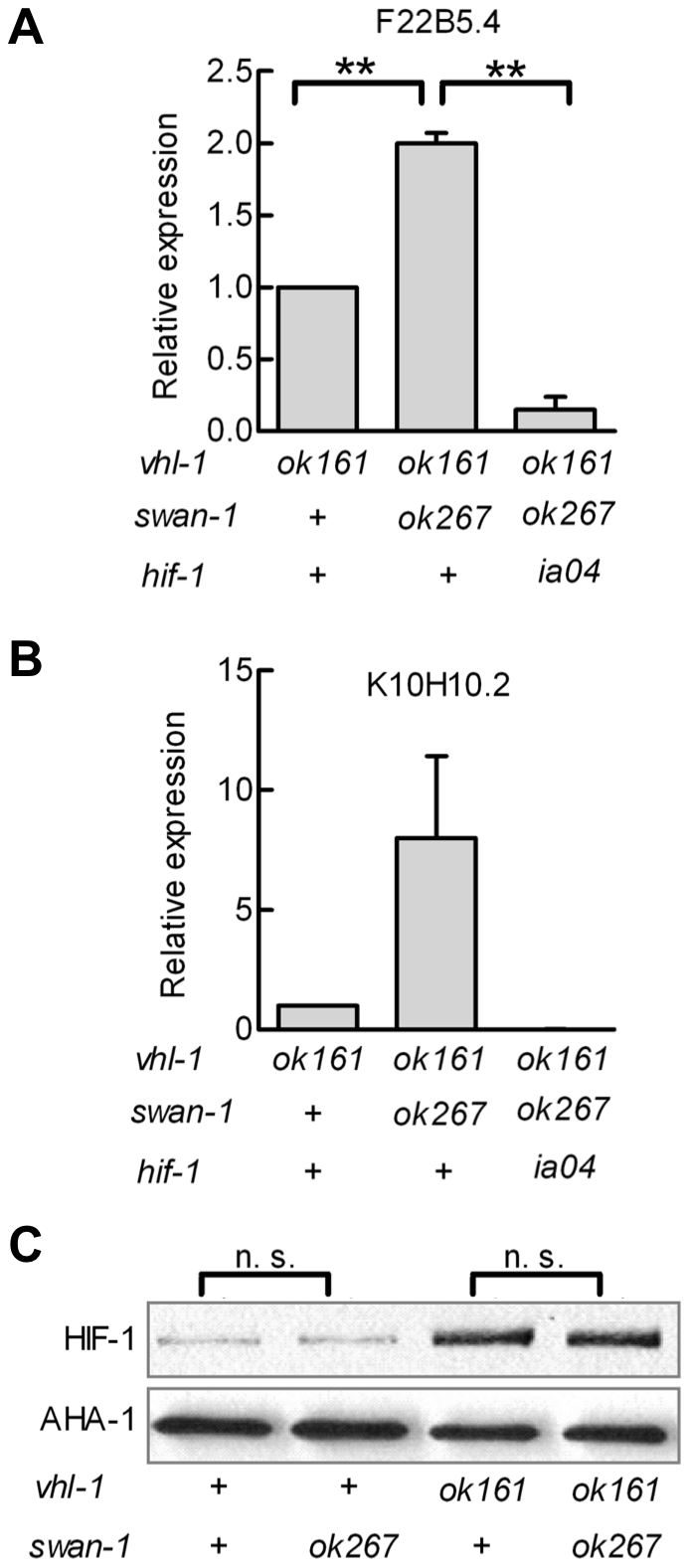
Loss-of-function mutations in *swan-1* increase expression of additional HIF-1 target genes, but do not alter HIF-1 protein levels. **A, B.** The expression levels of two HIF-1 target genes, F22B5.4 and K10H10.2, were assayed by quantitative RT-PCR. HIF-1 protein was stabilized by the *vhl-1(ok161)* mutation. For both genes assayed, the *swan-1(ok267)* mutation increased mRNA expression, and this phenotype was suppressed by the *hif-1(ia04)* loss-of-function mutation. The bars represent means from 3 independent replicates, and error bars reflect standard errors. **C.** The *swan-1(ok267)* mutation did not change HIF-1 protein levels, as assayed by protein blots in three biological replicates. n. s.: not significant; ** p<0.01.

Having established that *swan-1* negatively regulated P*nhr-57*::GFP expression, we next asked whether a strong loss-of-function mutation in *swan-1* also increased the expression of other HIF-1 target genes. Prior studies had demonstrated that K10H10.2 and F22B5.4 were induced by hypoxia in a *hif-1*-dependent manner and that they were over-expressed in *vhl-1, egl-9*, or *rhy-1* loss-of-function mutants [31], [32], [33], [39]. As shown in Figure 4A and 4B, mRNA levels for both K10H10.2 and F22B5.4 increased in *swan-1(ok267), vhl-1(ok161)* double mutants, relative to the *vhl-1(ok161)* single mutants. These data represent at least three biological replicates of realtime RT-PCR experiments. As shown in Figure 4C, the *swan-1* deletion mutation did not have a significant effect on HIF-1 protein levels.

### In the presence of stabilized HIF-1, a *swan-1* loss-of-function mutation protected *C. elegans* from *P. aeruginosa* fast killing

**Figure 5:**
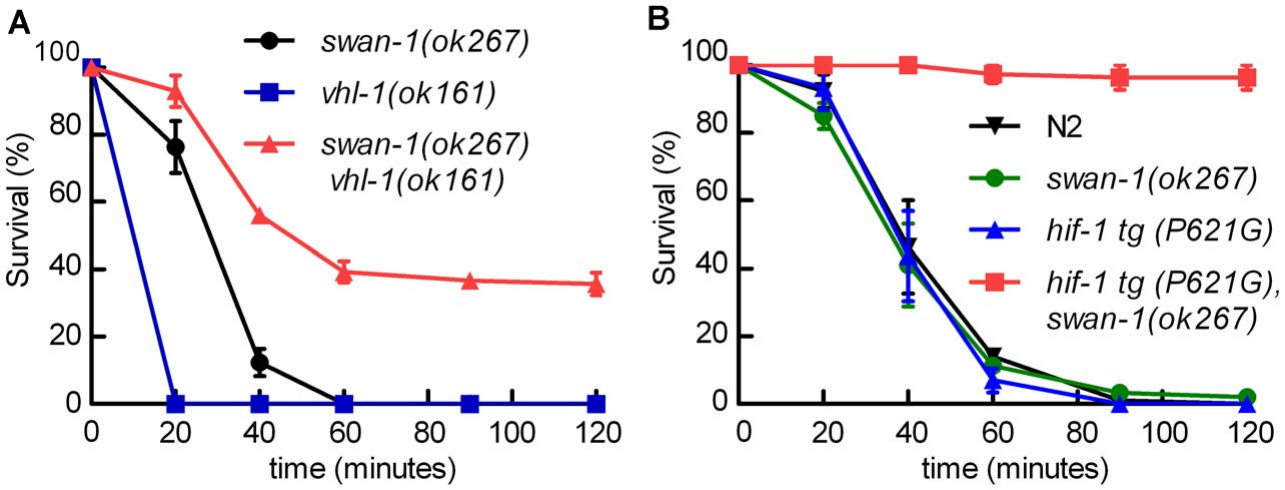
The *swan-1 (ok267), vhl-1 (ok161)* double mutant is more resistant to *P. aeruginosa* PAO1 fast killing than either single mutant. **A, B.** Percent *C. elegans* survival over time on *P. aeruginosa* PAO1. **A.** *vhl-1(ok161), swan-1(ok267)* double mutant animals were resistant to the fast killing by *P. aeruginosa,* PAO1 (∼40% survival after 2 hours). *vhl-1(ok161)* or *swan-1(ok267)* single mutants were not resistant. **B.** The *swan-1 (ok267)* mutation increased resistance to *P. aeruginosa* PAO1 fast killing in animals expressing stabilized HIF-1 protein. Each point on the line graph represents a mean from at least 3 replicate experiments. The error bars represent standard errors. These data are presented in tabular format in Text S1.

We hypothesized that the combination of HIF-1 stabilization and deletion of *swan-1* might result in a *P. aeruginosa* resistance phenotype similar to that of *egl-9* or *rhy-1* mutants. Stabilization of HIF-1, through either the stabilizing P621G mutation in HIF-1 transgenes or by mutation of the *vhl-1* E3 ligase, was not sufficient to protect *C. elegans* from *P. aeruginosa* PAO1 fast killing (Figure 1B and 5A). However, *swan-1(ok267), vhl-1(ok161)* double mutants were much more resistant than wild-type animals (>40% survived after two hours) (Figure 5A and Text S1). As expected, this resistance was suppressed by a *hif-1* loss-of-function mutation (Text S1). Similarly, in transgenic animals expressing HIF-1(P621G), the *swan-1(ok267)* mutation enabled almost 100% survival after two hours on a *P. aeruginosa* PAO1 lawn (Figure 5B). Similar results were obtained using an independent *hif-1(P621G)* transgenic line (Text S1).

### Interaction between the EGL-9 and SWAN-1 proteins

**Figure 6:**
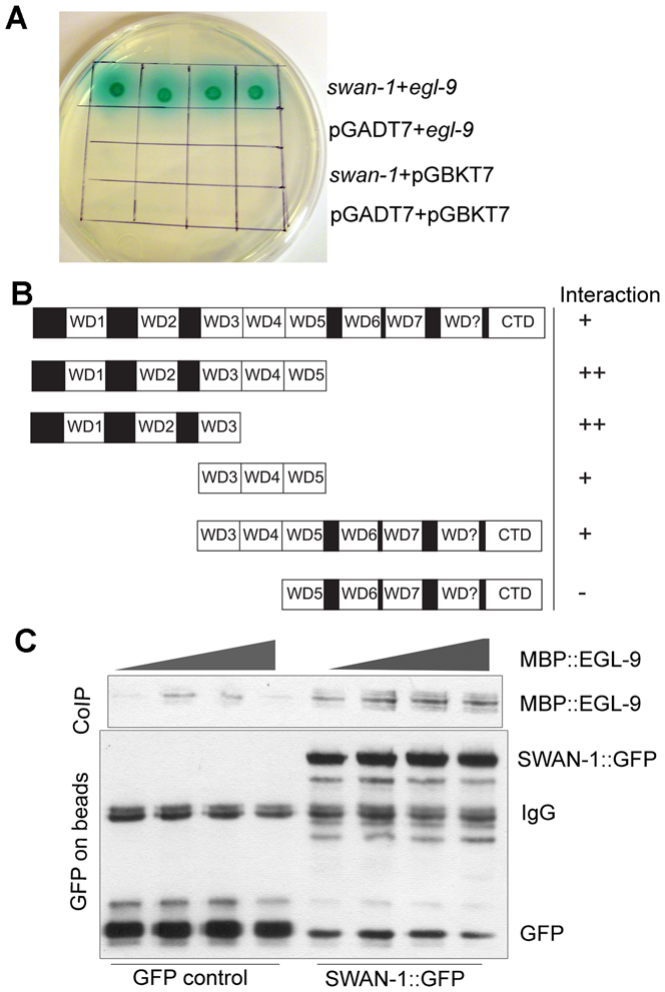
SWAN-1 binds to EGL-9. **A.** In yeast two-hybrid assays, SWAN-1 interacted with EGL-9, as assayed by growth on Ade^−^/His^−^/Leu^−^/Trp^−^ media and by α-galactosidase activity with 1×, 10×, 100×, 1000× dilution from left to right. In control experiments, yeast with the bait and prey vectors without inserts (pGADT7 and pGBKT7) did not grow in these conditions. **B.** SWAN-1 deletions were assayed for interaction with EGL-9 in the yeast two-hybrid system. The relative positions of seven evolutionary conserved WD domains, one degenerate WD domain (labeled WD?) and the C-terminal domain (CTD) are indicated. β-galactosidase activity and growth were scored in at least 3 replicates for each construct, and the strength of the interactions were assessed by comparison to the empty vector pGADT7 negative control (++: p<0.0001; +: 0.000<p<0.05, −: p>0.05). The amino-terminal domain of SWAN-1 containing 3 WD repeats interacted strongly with EGL-9. **C.** In additional interaction studies, the SWAN-1::GFP protein fusion or GFP was immunoprecipitated from transgenic worms with GFP-specific antibodies coupled to Sepharose beads and then incubated with bacterially-expressed EGL-9 fused to maltose binding protein (MBP::EGL-9). MBP::EGL-9 co-purified with SWAN-1::GFP (lanes 5–8). Negative controls using GFP are shown in lanes 1–4.

Since the genetic data suggested that SWAN-1 and EGL-9 acted in concert to inhibit HIF-1 activity, we next asked whether the two proteins interacted directly. To address this, we performed yeast-two-hybrid assays. In these assays, the EGL-9 catalytic domain was fused to the GAL4 DNA binding domain. In control experiments, this protein fusion by itself did not activate expression of reporter genes that were positively regulated by GAL4 upstream activating sequences. When the EGL-9 protein fusion was combined with a protein containing SWAN-1 fused to the GAL4 activation domain, the two proteins interacted to allow yeast growth on nutrient deficient plates (-Ade/-His/-Leu/-Trp) and to activate α-galactosidase expression (Figure 6A). To further define the regions of SWAN-1 that interacted with EGL-9, we assayed five SWAN-1 deletions, and these results are summarized in Figure 6B. A construct containing only the first three WD repeats of SWAN-1 was able to interact strongly with EGL-9 in yeast two-hybrid assays, whereas a construct that lacked the first four WD repeats did not interact with EGL-9.

To further test the hypothesis that EGL-9 and SWAN-1 could interact in a common complex, we conducted co-immunoprecipitation studies. In these experiments, the EGL-9 catalytic domain was fused to maltose binding protein and expressed in *E. coli*. A SWAN-1::GFP fusion protein was expressed in *C. elegans* and purified with a GFP-specific monoclonal antibody coupled to Sepharose beads. In control experiments, GFP alone was purified from worms. To assess interactions, the SWAN-1::GFP and MBP::EGL-9 proteins were co-incubated. Then, GFP-interacting proteins were isolated, and unbound proteins were washed away. As shown in Figure 6C, MBP::EGL-9 was coimmunoprecipitated with SWAN-1::GFP.

Prior studies had demonstrated that SWAN-1 interacted with Rac GTPases and the Rac effector UNC-115, and *swan-1* had been shown to repress Rac GTPase activity in neurons [38]. Thus, we considered models in which *swan-1* repressed HIF-1 activity by inhibiting Rac GTPases. However, depletion of *unc-115*, *rac-2, ced-10* or *mig-2* by mutation or RNAi did not abolish the induction of P*nhr-57::*GFP expression in *swan-1(ok267)*, *vhl-1(ok161)* animals (Text S1). This suggested that SWAN-1 had at least two functions: it interacted with Rac GTPases in neurons to regulate cell migration, and it inhibited HIF-1 transcriptional activity, probably through interaction with EGL-9.

### Mutation of *mbk-1*/DYRK suppresses the *swan-1* mutant phenotype

**Figure 7:**
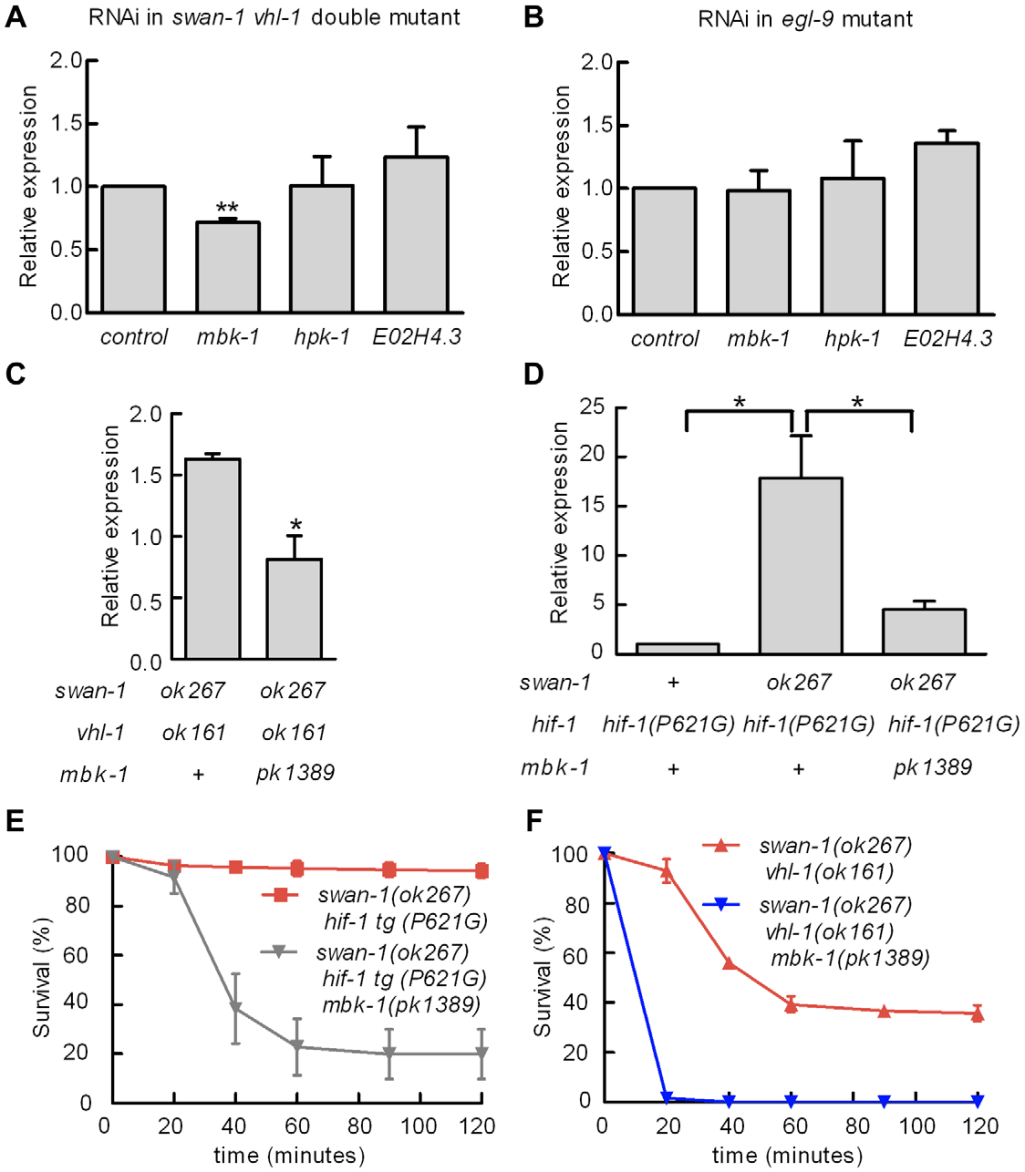
Genetic interactions between *swan-1* and *mbk-1*/DYRK to regulate P*nhr-57:*:GFP expression and resistance to *P. aeruginosa* fast killing. **A, B.** Bacterially mediated RNAi was used to decrease expression of individual DYRK homologs in *C. elegans*, and the effects on *Pnhr-57*::GFP expression was determined by protein blots. The bars show mean values from 3 replicate experiments; error bars represent standard errors. ** p<0.01. **A.** In *swan-1(ok267), vhl-1(ok161)* double mutants, *mbk-1* RNAi significantly decreased expression of the reporter, but RNAi for the other DYRK homologs did not change *Pnhr-57*::GFP levels relative to the empty vector control. **B.** In *egl-9(sa307)* animals, RNAi for the DYRK homologs tested had no effect on *Pnhr-57*::GFP. **C, D.** The *mbk-1(pk1389)* deletion mutation suppressed *Pnhr-57*::GFP expression in *swan-1(ok267), vhl-1(ok161)* double mutant animals (**C**) and in *swan-1(ok267)* animals expressing stabilized HIF-1(P621G) protein (**D**). **E, F.** The *mbk-1(pk1389)* mutation suppressed resistance to *P. aeruginosa* PAO1 in *swan-1(ok267)* animals. In these experiments, HIF-1 protein was stabilized by either the *vhl-1(ok161)* loss-of-function mutation (**E**) or the HIF-1(P621) stabilizing mutation (**F**). Each experiment was repeated at least three times with 30–50 animals for each strain. The error bars represent standard errors. *: p<0.05; **: p<0.01. These data are presented in tabular format in Text S1.

Homologs of SWAN-1 have been shown to interact with DYRK dual-specificity tyrosine-phosphorylation regulated kinases in yeast, zebrafish, and mammalian systems [42], [44]. We hypothesized that *swan-1* could interact with a DYRK homolog to regulate HIF-1 transcriptional activity. To test this, we used bacterially-mediated RNAi to knock down the expression of *mbk-1, hpk-1* and E02H4.3, three *C. elegans* genes homologous to mammalian DYRK genes. As shown in Figure 7A, *mbk-1* RNAi suppressed P*nhr-57::*GFP expression in *swan-1(ok267), vhl-1(ok161)* double mutants, while the other two RNAi treatments did not. Interestingly, *mbk-1* RNAi did not inhibit expression of the reporter in *egl-9* mutant animals (Figure 7B). We obtained similar results using the *mbk-1(pk1389)* loss-of-function mutation (Figure 7C). The *mbk-1* deletion allele also inhibited P*nhr-57::*GFP expression in *swan-1(ok267)* animals expressing the *hif-1(P621G)* transgene (Figure 7D).

We next asked whether *mbk-1* contributed to *swan-1*-mediated *P. aeruginosa* PAO1 resistance. As shown in Figure 7E and Text S1, the *mbk-1(pk1389)* mutation completely suppressed the PAO1 fast killing resistance phenotype in *swan-1(ok267), vhl-1(ok161)* double mutant animals. Further, *mbk-1(pk1389)* reduced PAO1 resistance in the *swan-1(ok267), hif-1(P621G)* genetic background, as assayed in two independently isolated *hif-1(P621G)* transgenic lines (Figure 7F and Text S1). Interestingly, *egl-9 mbk-1* double mutants are highly resistant to fast killing (Text S1). Thus, in assays of P*nhr-57*::GFP or *P. aeruginosa* PAO1 fast killing, the *mbk-1* mutation suppresses the *swan-1 vhl-1* double mutant phenotype, but not the *egl-9* loss-of-function phenotype.

## Discussion

### HIF-1 protects *C. elegans* from *P. aeruginosa* PAO1 fast killing

**Figure 8:**
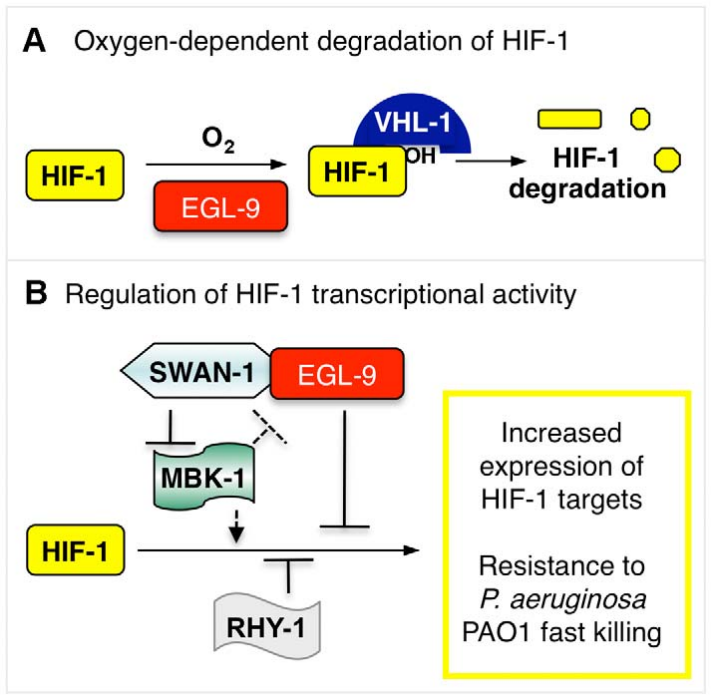
Model illustrating HIF-1 regulation. (**A**) The stability of HIF-1 protein is regulated by oxygen-dependent degradation. The EGL-9 enzyme hydroxylates HIF-1 protein in a reaction that requires molecular oxygen. Once modified, HIF-1 binds VHL-1, which promotes proteasomal degradation of HIF-1. (**B**) In addition to its role in controlling HIF-1 protein levels, EGL-9 represses HIF-1 transcriptional activity via VHL-1-independent pathway(s). SWAN-1 and RHY-1 also inhibit HIF-1-mediated transcription. MBK-1 acts downstream of SWAN-1 to promote HIF-1 activity. The substrates of the MBK-1 DYRK kinase are not yet known. Combinations of mutations that both stabilize HIF-1 protein and de-repress HIF-1 transcriptional activity cause increased resistance to *P. aeruginosa* PAO1 fast killing.

Mutations, alone or in combination, that dramatically increase HIF-1-mediated gene expression can protect *C. elegans* from *P. aeruginosa* PAO1 fast killing. Prior studies had discovered that loss-of-function mutations in *egl-9* conferred resistance to *P. aeruginosa* PAO1 fast killing and to cyanide poisoning, but the role of *hif-1* had not been investigated [27], [28]. Here, we establish that the resistance of *egl-9* mutants to this pathogen is dependent upon *hif-1* function (Figure 1A). EGL-9 is a bifunctional protein, and it regulates both HIF-1 protein stability and HIF-1 transcriptional activity [32], [33] (Figure 8). Interestingly, we find that stabilization of HIF-1 protein is not sufficient to protect *C. elegans* from *P. aeruginosa* fast killing, but mutations that disable both EGL-9 pathways confer resistance. We propose that over-expression of HIF-1 targets beyond a threshold level protects *C. elegans* from the cyanide produced by *P. aeruginosa* PAO1. It is also possible that mutation of *egl-9* allows HIF-1-mediated transcription in specific cells or tissues that are especially important to this resistance phenotype.

Hydrogen cyanide is an inhibitor of cytochrome c oxidase, and it is a potent toxin. The cyanide produced by *P. aeruginosa* in cystic fibrosis patients is recognized as a clinically important virulence factor [3]. Cyanide inhibits cytochrome c oxidase, severely disabling ATP synthesis through oxidative phosphorylation. Interestingly, *egl-9* mutant animals are also resistant to hydrogen sulfide, and H_2_S is also a cytochrome c oxidase inhibitor [45], [46]. A parsimonious explanation is that persistent over-expression of HIF-1 targets protects *C. elegans* from cyanide or hydrogen sulfide treatments that disable oxidative phosphorylation. These findings introduce an important question: which HIF-1 target genes protect *C. elegans* from *P. aeruginosa* PAO1 fast killing, cyanide exposure, and/or hydrogen sulfide? Prior studies have investigated the genes induced by short-term moderate hypoxia at 0.1% oxygen for 4 hours at room temperature [6]. Future studies will examine the changes in gene expression that are common to mutants or mutant combinations that activate HIF-1 and confer resistance to *P. aeruginosa.*

Increased expression of HIF-1 targets has been shown to protect *C. elegans* from diverse pathogens or stresses [30], [36], [37], [45], [47]. Bellier *et al.* isolated a loss-of-function mutation in *egl-9* in a screen for mutations that protected *C. elegans* from pore-forming toxins [30]. *egl-9* mutants have also been shown to be resistant to enteropathogenic *E. coli* E2348/69 [47]. Additionally, mutations that stabilize HIF-1 or increase expression of HIF-1 targets have been shown to increase *C. elegans* resistance to polyglutamine or beta-amyloid toxicity and heat stress [30], [34], [36], [37]. It is not yet known whether the same HIF-1 targets mediate all of these resistance phenotypes, but we anticipate that each of these functions may require multiple direct and indirect HIF-1 targets.

### SWAN-1, a novel regulator of HIF-1

SWAN-1 is an evolutionarily conserved WD-repeat protein of the AN11 family [38]. The data presented here show that SWAN-1 represses HIF-1-mediated gene expression, but does not control HIF-1 protein levels. While *swan-1* RNAi does increase expression of the P*nhr-57*:GFP reporter in an otherwise wild-type background (Figure 3C), loss of *swan-1* function alone is not sufficient to confer resistance to cyanide released by *P. aeruginosa* PAO1 (Figures 5A and 5B). Resistance to fast killing requires a second mutation that protects HIF-1 protein from oxygen-dependent degradation. Double mutants that carry a loss-of-function mutation in *swan-1* and a mutation that stabilizes HIF-1 protein are phenotypically similar to *egl-9* loss-of-function mutants, as assayed by fertility, egg laying defects, over-expression of HIF-1 targets, and resistance to *P. aeruginosa* fast killing (Figures 1A, 2G, 4A, 4B, 5A). *swan-1, vhl-1* double mutants also exhibit increased resistance to hydrogen cyanide (unpublished data). Importantly, we show that SWAN-1 forms a complex with EGL-9 (Figure 6A, 6C). Collectively, these data suggest that SWAN-1 and EGL-9 interact directly to repress HIF-1 transcriptional activity.

The AN11 family is evolutionarily conserved, and comparative studies may provide important insights to the roles of these proteins in stress resistance and transcriptional regulation. Petunia AN11 interacts with a MYB family transcription factor, and the human HAN11 gene has been shown to partially rescue the Petunia *an11* mutant phenotype [43]. In human cells, zebrafish, and in yeast, AN11 homologs have been shown to form complexes with DYRK kinases [42], [44], [48]. There are five DYRK members in mammals: DYRK1A, DYRK1B, DYRK2, DYRK3 and DYRK4 [49]. Of these, DYRK1A has been characterized most extensively, and it is associated with Down Syndrome [50], [51], [52]. DYRK1A is a multifunctional protein and has more than two dozen targets or interacting proteins, including GLI1, STAT3, and eIF2Bε [49]. HAN11 was shown to decrease DYRK1A-mediated phosphorylation of GLI1 in a HEK293T cell line [53].

The genetic analyses presented here suggest that in the absence of SWAN-1, MBK-1/DYRK activates HIF-1 (illustrated in Figure 8). Specifically, a loss-of-function mutation in *mbk-1* suppresses the *swan-1, vhl-1* double mutant phenotypes, as assayed by expression of P*nhr-57::*GFP expression and by *P. aeruginosa* PAO1 fast killing (Figure 7). These genetic data suggest that *mbk-1* acts downstream of *swan-1*. Interestingly, mutation of *mbk-1* does not suppress the *egl-9* mutant phenotype (Text S1). There are at least two models that could explain these findings. First, EGL-9-mediated repression of HIF-1 transcriptional activity may be modulated by SWAN-1 and MBK-1 without being totally dependent upon these regulators. An alternative model is that MBK-1 and SWAN-1 act in parallel to EGL-9 to repress HIF-1 activity. While we favor the first model, we recognize both possibilities in Figure 8. A goal for future studies will be to identify the targets of MBK-1/DYRK to better understand how MBK-1 promotes HIF-1 activity.

A long-term goal will be to understand how hypoxia-induced gene expression influences the progression of *P. aeruginosa* infections. *P. aeruginosa* can survive in anaerobic environments, and the formation of biofilms likely restricts oxygen availability to infected tissues in human patients. Our findings in *C. elegans* suggest that pharmacological inhibitors of the HIF prolyl hydroxylases might contribute to combinatorial therapies to protect cells from the cyanide produced by *P. aeruginosa* PAO1.

## Materials and Methods

### Alleles and worm culture

*C. elegans* were grown at 20°C using standard methods, unless other culture conditions are specified [54]. The loss-of-function alleles and transgenic lines used in this study are listed in Text S1. The *swan-1(ok267)* mutant allele was backcrossed to wild-type animals three times prior to phenotypic analyses [38].

### Mutagenesis

The EMS forward genetic screen was performed as described previously [33]. Briefly, the parental strain carrying P*nhr-57::GFP* and *vhl-1(ok161)* was mutagenized with EMS, and the F2 progeny were screened for increased expression of the P*nhr-57*::GFP reporter using fluorescent stereomicroscopy. We generated the *rhy-1* loss-of-function alleles *ia59, ia62, ia63, ia64* in a screen for Mos1 transposon-mediated mutations that caused P*nhr-57*::GFP overexpression. The methods for Mos1 mobilization have been described previously [32], [55].

### Mapping *ia50* to the *swan-1* locus

The *ia50* mutant allele was out-crossed twice to the parental strain prior to any further mapping or characterization. Chromosome and interval mapping were performed as described previously using single-nucleotide polymorphisms (SNPs) between the Bristol N2 and Hawaiian strains [56]. Briefly, the *iaIs07 (*P*nhr-57*::GFP*)* transgene and the *vhl-1(ok161)* mutation were crossed extensively into the Hawaiian genetic background. The resulting males were crossed to *ia50 vhl-1(ok161)* double mutants carrying the *iaIs07(*P*nhr-57*::GFP*)* marker. Fifty F2 animals exhibiting the *vhl-1, ia50* double mutant phenotype and fifty animals exhibiting the *vhl-1 (ok161)* single mutant phenotype (intestinal GFP expression) were picked into separate tubes, and genomic DNA was prepared. Analyses of the divergent SNPs showed enrichment of Bristol bands in mutant lanes and an enrichment of Hawaiian bands in non-mutant lanes for SNPs lying between -5 and +13 mu on chromosome V. For interval mapping, individual self-progeny of F1 hermaphrodites (described above) with the double mutant phenotype were picked into a 96-well plate to prepare the genomic DNA, and four SNPs were analyzed (−5, +1, +6, +13) [56]. We then used two SNPs, *R10D6* (+5.83 mu) and *pkP5086* (+6.42 mu), to do three point mapping of the *ia50* mutation.

### RNA interference

RNAi was performed as previously described [34]. Bacterial strains containing RNAi constructs were purchased from Geneservice Ltd., and inserts were validated by sequencing.

### Fertility assays

Individual L4-stage worms were placed on NGM plates with fresh OP50 bacterial food. The worms were transferred onto fresh plates every 12 hours, and the total progeny laid on each plate was counted and recorded at each time point. This procedure was continued until the worms reached the end of their reproductive capacity.

### MBP::EGL-9 protein expression

To build pSZ18, the bacterial expression vector for EGL-9::MBP, *egl-9* cDNA was amplified with two primers: 5′GGTGGATCCAAACCAACGGTATCCAGAAC and 5′GGGATCCGATGTAATACTCTGGGTTTGTGG. The PCR products were cut with *Bam*HI and *Pst*I and ligated into the pMal-p2x vector (from New England Biolabs). The plasmid expressing EGL-9 fused to maltose binding protein was transformed into BL21 (DE3) bacteria, and a single colony was inoculated into liquid media and cultured for 16 hours at 37°C. The bacterial culture was diluted by 1:200 to 50 ml and cultured for 2.5 hours. IPTG was added to a final concentration of 1 mM. After 4 hours, the bacteria were pelleted and frozen in liquid nitrogen. The bacteria were frozen at −80°C overnight before adding bacterial lysis buffer (50 mM Tris PH 7.5, NaCl 150 mM, NP-40 0.1%), 0.25 mg/ml lysozyme, 0.01 mg/ml DNase, and 1× protease inhibitor cocktail (from Roche). After four hours at 4°C, the lysate was centrifuged at 12000 g for 20 min and the supernatant was kept for further use.

### *P. aeruginosa* fast killing assays

The fast killing assay follows the approach described previously [27], [28]. Briefly, a single *P. aeruginosa* PAO1 colony was inoculated and cultured at 37°C for 16 hours in 3–5 ml brain heart infusion (BHI) broth. The culture was then diluted 100-fold. 300 µl of diluted bacteria was evenly spread on 60 mm diameter Petri dishes with 7 ml BHI agar. To minimize the possibility of animals escaping from the bacterial lawn, bacteria were spread to cover the whole plate. The plates were incubated at 37°C for 24 hours and then cooled to room temperature for 30 minutes. Thirty to fifty developmentally synchronized L4-stage *C. elegans* were put on the lawn and incubated at room temperature (22°C). Lids to the petri plates remained closed during this time to keep hydrogen cyanide from evaporating. *C. elegans* were scored as paralyzed or dead if they did not move when the plate was tapped against the microscope stage. Each data point represents at least three independent replicate experiments.

### Protein blots

The methods for protein blots have been described previously [32]. At least three biological replicates were analyzed for each experiment. Transgenic strains expressing epitope-tagged HIF-1 were used to assay HIF-1 protein levels and each lane of a protein gel included lysate from 40–100 L4-stage animals. For the P*nhr-57*::GFP reporter, we used 5–50 L4 animals. The statistical significance of differences was assessed by two-sample paired t-tests or one way ANOVA with Bonferroni post test.

### Real time PCR

The methods for real time PCR were as described previously [32]. At least three biological replicates were analyzed for each experiment, and each PCR reaction was performed in duplicate. The statistical significance of differences was assessed by two-sample paired t-tests or one way ANOVA with Bonferroni post test.

### Yeast two-hybrid assays

For yeast two-hybrid experiments, *C. elegans* cDNA sequences were inserted in to the pGBKT7 and pGADT7 vectors from Clonetech. The sequences encoding the EGL-9 catalytic domain were amplified with two primers, *egl-9-*F: CCGGAATTCGGTCTCGCACTAAGCATTCACC and *egl-9-*R: CGCGGATCCCCGTGGT CTCAAAAGTGATCCAAT, and cloned into pGBKT7 (GAL4 DNA-binding domain vector). This construct did not result in detectable autoactivation. Dr. Erik Lundquist kindly provided the plasmid which expressed SWAN-1 fused to the GAL4 transcriptional activation domain. The deletion vectors (shown in Figure 6B) were amplified from this *swan-1* prey plasmid [38]. The primer sets are listed in Text S1.

The AH109 yeast strain was used. Transformations of plasmids into yeast were performed as described previously [57], [58]. The negative controls included the co-transformation of pGADT7-*swan-1* plasmids with pGBKT7 (empty vector); the co-transformation of pGBKT7-*egl-9* and pGADT7; and the co-transformation of pGBKT and pGADT7. To test for interactions, yeast colonies carrying both plasmids (selected on Leu^−^, Trp^−^ plates) were cultured in 2 ml Leu^−^/Trp^−^ liquid SD-medium for 24 hours at 30°C. Twenty individual colonies were then assayed for growth on Ade^−^/His^−^/Leu^−^/Trp^−^ X-α-gal SD-medium plates. Additionally, six Leu^+^/Trp^+^ colonies were cultured for quantification of the β-galactosidase activity using O-nitrophenyl-B-D-galactopyranoside as a substrate.

### Co-immunoprecipitation experiments

The *C. elegans* strain expressing SWAN-1::GFP [transgenic array *lqEx19 (Pswan-1:: swan-1::gfp)*] was generously provided by Erik Lundquist. *C. elegans* were grown on 100 mm enriched media plates with NA22 bacteria, and 0.4 ml mixed-stage worms were harvested. Animals were washed with cold M9 buffer three times and were washed once with worm lysis buffer (50 mM Tris-HCl PH 7.4, 150 mM NaCl, 0.5% Triton-X-100, 10% glycerol, 1 mM DTT, and 1X protease inhibitor cocktail from Roche). The worm pellet was resuspended in 1.2 ml worm lysis buffer, and the animals were lysed in a french press (Thermo Electron Corporation) three times at 1000 psi. To perform immunoprecipitations, 80 µl G-Sepharose beads were washed with worm lysis buffer three times for 10 minutes each, and they were then incubated with 80 µl 0.4 mg/ml GFP antibody (Roche) overnight. Beads were washed with worm lysis buffer three times for 5 minutes and divided into two 40 µl aliquots, each of which was incubated with 600 µl of SWAN-1::GFP or GFP worm lysate at 4°C for 4 hours. Beads with either control GFP [*Pnhr-57*::GFP] or SWAN-1::GFP were washed (three times 10 minutes in cold lysis buffer) and incubated with MBP::EGL-9. After four hours incubation at 4°C, beads were washed with worm lysis buffer and boiled in 40 µl 1xSDS buffer. The proteins were fractionated by 10% SDS polyacrylamide gel electrophoresis, and blots were probed with maltose binding protein rabbit antiserum (from NEB at 1∶3000 dilution) or mouse GFP monoclonal antibody (clones 7.1 and 13.1 from Roche at 1∶1000 dilution).

## Supporting Information

Text S1 Supplemental data tables
(0.54 MB DOC)
