# Supplementary material for: C. elegans SWAN-1 Binds to EGL-9 and Regulates HIF-1-Mediated Resistance to the Bacterial Pathogen Pseudomonas aeruginosa PAO1
Source: PLoS Pathog. 2010 Aug 26;6(8):e1001075. doi: 10.1371/journal.ppat.1001075 (PMC2928816; doi:10.1371/journal.ppat.1001075)
Supplement: Text S1 — Supplemental data tables (0.54 MB DOC) [file ppat.1001075.s001.doc]

SUPPLEMENTAL MATERIALS

**Contents:**

**Table S1.** Data for survival of wildtype N2*, hif-1(ia04), egl-9(sa307),* and *egl-9(sa307) hif-1(ia04)* *C. elegans* on *Pseudomonas aeruginosa* PAO1 over time.

**Table S2.** Data for survival of wildtype N2*, hif-1(ia04), egl-9(sa307),* and *egl-9(sa307) hif-1(ia04), rhy-1(ok1402)* double mutant after four hours on MP507 bacteria.

**Table S3.** Data for survival of wildtype N2 and transgenic strains expressing *hif-1* or *hif-1(P621G)* on *Pseudomonas aeruginosa* PAO1 over time

**Table S4.** Survival data for *swan-1(ok267), vhl-1(ok161), vhl-1(ok161) swan-1(ok267)* double and *vhl-1(ok161) swan-1(ok267)* *mbk-1(pk1389)* triple mutant *C. elegans* on *Pseudomonas aeruginosa* PAO1 over time

**Table S5.** Molecular characterization of new *rhy-1* alleles

**Table S6.** Survival data for *swan-1(ok267), vhl-1(ok161), vhl-1(ok161) swan-1(ok267)* double and *vhl-1(ok161) swan-1(ok267)* *hif-1(ia04)* triple mutant *C. elegans* after four hours on *Pseudomonas aeruginosa* PAO1 or MP507

**Table S7.** Survival percentages for N2, *swan-1(ok267), hif-1(P621G)* transgenic strains, and *swan-1(ok267)* with transgene *hif-1(P621G)* on *Pseudomonas aeruginosa* PAO1 lawn over time

**Table S8.** Survival percentages for the following *C. elegans* strains on *P. aeruginosa* PA01 over time: *mbk-1(pk1389), swan-1(ok267)* with *hif-1(P621G)* transgene, and *swan-1(ok267) mbk-1(pk1389)* with transgene *hif-1(P621G)*

**Table S9.** Survival percentages for the following *C. elegans* strains on *P. aeruginosa* PA01 over time: *mbk-1(pk1389), egl-9(sa307), egl-9(sa307) mbk-1(pk1389)* double mutant*,* *rhy-1(ia59) mbk-1(pk1389)*

**Table S10.** Protein-protein interaction between SWAN-1 domains and EGL-9 in yeast two-hybrid assays

**Table S11.** Primers for making *swan-1* deletion constructs

**Table S12.** Alleles used in this study

**Table S13**. Transgenic *C. elegans* used in this study and references

**Figure S1.** Inhibition of *unc-115, mig-2,* or *ced-10* function by RNAi or mutation did not change P*nhr-57::*GFP expression in *vhl-1, vhl-1 swan-1*, or *egl-9* mutant animals

**References**

**Table S1**. Data for survival of wildtype N2*, hif-1(ia04), egl-9(sa307),* and *egl-9(sa307) hif-1(ia04)* *C. elegans* on *Pseudomonas aeruginosa* PAO1 over time.

|  | 0min | 20min | 40min | 60min | 90min | 120min | Replicates/ total animals |
| --- | --- | --- | --- | --- | --- | --- | --- |
| N2 | 100% | 84%±4% | 16%±6% | 9%±2% | 0% | 0% | 3/102 |
| *hif-1(ia04)* | 100% | 90%±4% | 24%±16% | 6%±7% | 0% | 0% | 3/116 |
| *egl-9(sa307)* | 100% | 100% | 100% | 100% | 100% | 100% | 3/109 |
| *egl-9(sa307)*  *hif-1(ia04)* | 100% | 93%±4% | 19%±8% | 4%±5% | 1%±2% | 0% | 3/107 |

**Table S2**. Data for survival of wildtype N2*, hif-1(ia04), egl-9(sa307),* and *egl-9(sa307) hif-1(ia04), rhy-1(ok1402)* double mutant after four hours on MP507 bacteria *

| Genotype | Average | Replicates/ total animals |
| --- | --- | --- |
| N2 | 96.9%±4% | 2/74 |
| *egl-9(sa307)* | 100.0% | 2/70 |
| *egl-9(sa307) hif-1(ia04)* | 98.7%±2% | 3/116 |
| *rhy-1(ok1402)* | 97.3%±2% | 4/150 |

* MP507 is a *P. aeruginosa* strain carrying a mutation in the *hcnC* gene, which encodes a subunit of hydrogen cyanide synthase (Gallagher and Manoil 2001)

**Table S3.** Data for survival of wildtype N2 and transgenic strains expressing *hif-1* or *hif-1(P621G)* on *Pseudomonas aeruginosa* PAO1 over time

|  | 0min | 20min | 40min | 60min | 90min | 120min | Replicates  / total animals |
| --- | --- | --- | --- | --- | --- | --- | --- |
| N2 | 100% | 80%±8% | 20%±7% | 4%±2% | 0% | 0% | 3/128 |
| *iaIs28** | 100% | 68%±20% | 13%±6% | 0% | 0% | 0% | 3/113 |
| *iaIs32#* | 100% | 85%±9% | 34%±22% | 3%±3% | 0% | 0% | 3/117 |
| *iaIs33#* | 100% | 58%±19% | 5%±3% | 0% | 0% | 0% | 3/114 |
| *iaIs34#* | 100% | 67%±17% | 5%±2% | 0% | 0% | 0% | 3/113 |

*: *hif-1(+)* transgenic line; #: *hif-1(P621G)* transgenic lines

**Table S4.** Survival data for *swan-1(ok267), vhl-1(ok161), vhl-1(ok161) swan-1(ok267)* double and *vhl-1(ok161) swan-1(ok267)* *mbk-1(pk1389)* triple mutant *C. elegans* on *Pseudomonas aeruginosa* PAO1 over time

|  | 0min | 20min | 40min | 60min | 90min | 120min | Replicates/ total animals |
| --- | --- | --- | --- | --- | --- | --- | --- |
| *swan-1(ok267)* | 100% | 74%±6% | 13%±3% | 2%±2% | 2%±2% | 2%±2% | 4/156 |
| *vhl-1(ok161)* | 100% | 0% | 0% | 0% | 0% | 0% | 3/111 |
| *swan-1(ok267),*  *vhl-1(ok161)* | 100% | 93%±5% | 56%±2% | 39%±3% | 37%±3% | 36%±3% | 3/117 |
| *swan-1(ok267),*  *vhl-1(ok161),*  *mbk-1(pk1389)* | 100% | 16%±14% | 0% | 0% | 0% | 0% | 4/153 |

**Table S5.** Molecular characterization of new *rhy-1* alleles

| Alleles | Left flanking sequence | right flanking sequence | MOS1 landed in |
| --- | --- | --- | --- |
| ia59 | gaaactcgatg | tattggatatc | second exon |
| ia62 | aaatgcattta | ttcctgatact | first exon |
| ia63 | acacggcagta | ttccgatctgt | third exon |
| ia64 | tatctccttca | tatccagttgt | fourth exon |

**Table S6.** Survival data for *swan-1(ok267), vhl-1(ok161), vhl-1(ok161) swan-1(ok267)* double and *vhl-1(ok161) swan-1(ok267)* *hif-1(ia04)* triple mutant *C. elegans* after four hours on *Pseudomonas aeruginosa* PAO1 or MP507

|  | PAO1 | | MP507 | |
| --- | --- | --- | --- | --- |
|  | Survival rate | Replicates/ total animals | Survival rate | Replicates/ total animals |
| *swan-1(ok267)* | 0% | 4/194 | 100% | 3/115 |
| *vhl-1(ok161)* | 0% | 4/201 | 92%±5% | 3/130 |
| *swan-1(ok267)*  *vhl-1(ok161)* | 45%±4% | 5/251 | 98%±1% | 4/153 |
| *swan-1(ok267) hif-1(ia04)*  *vhl-1(ok161)* | 0% | 4/220 | 100% | 4/173 |

**Table S7.** Survival percentages for N2, *swan-1(ok267), hif-1(P621G)* transgenic strains, and *swan-1(ok267)* with transgene *hif-1(P621G)* on *Pseudomonas aeruginosa* PAO1 lawn over time

|  | 0min | 20min | 40min | 60min | 90min | 120min | Replicates  / total animals |
| --- | --- | --- | --- | --- | --- | --- | --- |
| N2 | 100% | 94%±4% | 54%±13% | 17%±3% | 4%±3% | 0% | 4/145 |
| *swan-1(ok267)* | 100% | 89%±5% | 50%±12% | 16%±5% | 8%±5% | 7%±5% | 4/147 |
| *iaIs32#* | 100% | 95%±5% | 52%±13% | 10%±4% | 0% | 0% | 4/160 |
| *swan-1(ok267,*  *iaIs32#* | 100% | 100% | 99%±1% | 96%±2% | 95%±3% | 95%±3% | 4/153 |
| *iaIs33#* | 100% | 94%±5% | 10%±3% | 2%±2% | 0% | 0% | 3/121 |
| *swan-1(ok267),*  *iaIs33#* | 100% | 100% | 100% | 100% | 100% | 100% | 4/156 |

#: *hif-1(P621G)* transgenic lines

**Table S8.** Survival percentages for the following C. elegans strains on P. aeruginosa PA01 over time: *mbk-1(pk1389), swan-1(ok267)* with *hif-1(P621G)* transgene, and *swan-1(ok267) mbk-1(pk1389)* with transgene *hif-1(P621G)*

|  | 0min | 20min | 40min | 60min | 90min | 120min | Replicates/ total animals |
| --- | --- | --- | --- | --- | --- | --- | --- |
| *mbk-1(pk1289)* | 100% | 69%±9% | 24%±13% | 2%±2% | 0% | 0% | 3/107 |
| *iaI32# swan-1(ok267)* | 100% | 97%±2% | 96%±3% | 95%±3% | 95±3% | 94%±3% | 5/185 |
| *iaIs33# swan-1(ok267)* | 100% | 100% | 99%±1% | 99%±1% | 99±1% | 99%±1% | 5/178 |
| *iais32# swan-1(ok267)*  *mbk-1(pk1389)* | 100% | 89%±7% | 35%±10% | 18%±7% | 16±6% | 16%±6% | 5/194 |
| *iaIs33# swan-1(ok267)*  *mbk-1(pk1389)* | 100% | 97%±1% | 42%±14% | 22%±12% | 20±10% | 20%±10% | 5/190 |

#: *hif-1(P621G)* transgenic lines

**Table S9.** Survival percentages for the following *C. elegans* strains on *P. aeruginosa* PA01 over time: *mbk-1(pk1389), egl-9(sa307), egl-9(sa307) mbk-1(pk1389)* double mutant*,* *rhy-1(ia59) mbk-1(pk1389)*

|  | 0min | 20min | 40min | 60min | 90min | 120min | Replicates/ total animals |
| --- | --- | --- | --- | --- | --- | --- | --- |
| *mbk-1(pk1389)* | 100% | 69%±9% | 24%±13% | 2%±2% | 0% | 0% | 3/107 |
| *egl-9(sa307)* | 100% | 100% | 100% | 100% | 100% | 100% | 3/109 |
| *egl-9(sa307)*  *mbk-1(pk1389)* | 100% | 100% | 100% | 100% | 100% | 100% | 3/110 |
| *rhy-1(ia59)*  *mbk-1(pk1389)* | 100% | 100% | 100% | 99%±1% | 99%±1% | 97%±1% | 3/92 |

**Table S10.** Protein-protein interaction between SWAN-1 domains and EGL-9 in yeast two-hybrid assays.

| Bait protein | Prey protein | Growth on  Selective medium^(a)^ | β-Galctosidase Activity  (Mean±SD, n=6) | Fold increase in β-Gal activity ^(b)^ |
| --- | --- | --- | --- | --- |
| EGL-9 | SWAN-1: full length (1-388aa) | + | 0.216±0.059 | 1.83 |
| EGL-9 | SWAN-1: ATG-WD5(1-260aa) | ++ | 0.365±0.076 | 3.09 |
| EGL-9 | SWAN-1: ATG-WD3 (1-196aa) | ++ | 0.494±0.058 | 4.19 |
| EGL-9 | SWAN-1: WD3-CTD (162-388aa) | + | 0.219±0.046 | 1.85 |
| EGL-9 | SWAN-1: WD3-WD5 (162-260aa) | + | 0.224±0.032 | 1.90 |
| EGL-9 | SWAN-1: WD5-CTD (227-388) | - | 0.131±0.039 | 1.11 |

(a). Yeast colonies on selective medium (SD medium lacking tryptophan, leucine, histidine, and adenine adding X-α-gal) within 3 days (++), 5 days (+) or no growth after 7 days (-).

(b). Six Leu^+^/Trp^+^ colonies of each pair of interactions were cultured for quantification of β-galactosidase activity using O-nitrophenyl-B-D-galctopyranoside as a substrate. β-galactosidase activity was expressed in 1 Miller Unit=1000хOD420/(tхVхOD600), where t is elapsed time of incubation in minutes, V is volume of the culture used, OD420 represents the absorbance of O-nitrophenol, and OD600 is the cell density of the culture. Fold increase over β-Galctosidase activity units of the strains expressing both empty prey and bait vectors.

**Table S11** Primers for making *swan-1* deletion constructs

| Constructs | Primer pairs* |
| --- | --- |
| Full length | swan-F1/ Swan-Rctd |
| N-terminus-WD5 | swan-F1/ Swan-Rwd5 |
| N-terminus-WD3 | swan-F1/ Swan-Rwd3 |
| WD3-WD5 | Swan-WD3F/ Swan-Rwd5 |
| WD3-C-terminus | Swan-WD3F/ Swan-Rctd |
| WD5-C-terminus | Swan-WD5F/ Swan-Rctd |

* Swan-F1: CCGGAATTCATGGCTACCAATGGTCACGCAGTG

Swan-WD3F: CCGGAATTCTCACAATATGGATCAGCTTTG

Swan-WD5F: CCGGAATTCTTTGCTAAAATCAATGGCGGT

Swan-Rctd: CGCATCGATAACTCGAAGAATCTCAAGAGT

Swan-Rwd5: CGCATCGATTTCATAGACAATTGTCGAGTG

Swan-Rwd3: CGCATCGATCTCAACATCATAAATTGTGCG

**Table S12.** Alleles that have been used in this study

| Genes | Alleles | Description | References |
| --- | --- | --- | --- |
| *hif-1* | *ia04* | Loss of function | (Jia*ng et a*l. 2001) |
| *vhl-1* | *ok161* | Loss of function | (Epste*in et a*l. 2001) |
| *egl-9* | *sa307* | Loss of function | (Dar*by et a*l. 1999) |
|  | *ia58* | Loss of function | (Sh*ao et a*l. 2009) |
|  | *ia60* | Loss of function | (Sh*ao et a*l. 2009) |
| *rhy-1* | *ok1402* | Loss of function | (Sh*en et a*l. 2006) |
| *swan-1* | *ok267* | Loss of function | (Ya*ng et a*l. 2006) |
|  | *ia50* | Loss of function | This study |
| *mbk-1* | *pk1389* | Loss of function | (Rai*ch et a*l. 2003) |

**Table S13**. Transgenic *C. elegans* used in this study and references.

| Transgenic lines | Description | References |
| --- | --- | --- |
| iaIs28* | *Phif-1::hif-1::HA-MYC-STREP* | (Sh*ao et a*l. 2009; Zha*ng et a*l. 2009) |
| iaIs32* | *Phif-1::hif-1(P621G)::MYC* |
| iaIs33* | *Phif-1::hif-1(P621G)::MYC* |
| iaIs34* | *Phif-1::hif-1(P621G)::MYC* |
| iaIs07* | *Pnhr-57::GFP* | (Sh*en et a*l. 2006) |
| lqEx19# | *Pswan-1::swan-1::GFP* | (Ya*ng et a*l. 2006) |

* *unc-119* was used as coinjection marker

# *lin-15* was used as coinjection marker (Yang, 2006, #56)

**Figure S1** Inhibition of *unc-115, mig-2,* or *ced-10* function by RNAi or mutation did not change P*nhr-57::*GFP expression in *vhl-1, vhl-1 swan-1*, or *egl-9* mutant animals.

A. Mutation of *unc-115* did significantly change *Pnhr-57*::GFP expression. B. *ced-10* or *mig-2* RNAi treatments did not alter *Pnhr-57*::GFP expression. C. Effects of loss-of-function mutations in *unc-115* or *mig-2* on *Pnhr-57*::GFP expression. D. Effects of loss-of-function mutations in *unc-115* or *mig-2* on *Pnhr-57*::GFP expression in *egl-9* mutants.

**References for methods, strains, and materials for supplemental data**

Darby, C., C. L. Cosma, J. H. Thomas and C. Manoil, 1999 Lethal paralysis of *Caenorhabditis elegans* by P*seudomonas aeruginosa*. Proc Natl Acad Sci U S A 96: 15202-15207.

Epstein, A. C., J. M. Gleadle, L. A. McNeill, K. S. Hewitson, J. O'Rourke *et al.*, 2001 C. elegans EGL-9 and mammalian homologs define a family of dioxygenases that regulate HIF by prolyl hydroxylation. Cell 107: 43-54.

Gallagher, L. A., and C. Manoil, 2001 *Pseudomonas aeruginosa* PAO1 kills *Caenorhabditis elegans* by cyanide poisoning. J Bacteriol 183: 6207-6214.

Jiang, H., R. Guo and J. A. Powell-Coffman, 2001 The *Caenorhabditis elegans hif-1* gene encodes a bHLH-PAS protein that is required for adaptation to hypoxia. Proc Natl Acad Sci U S A 98: 7916-7921.

Raich, W. B., C. Moorman, C. O. Lacefield, J. Lehrer, D. Bartsch *et al.*, 2003 Characterization of *Caenorhabditis elegans* homologs of the Down syndrome candidate gene DYRK1A. Genetics 163: 571-580.

Shao, Z., Y. Zhang and J. A. Powell-Coffman, 2009 Two distinct roles for EGL-9 in the regulation of HIF-1-mediated gene expression in *Caenorhabditis elegans*. Genetics 183: 821-829.

Shen, C., Z. Shao and J. A. Powell-Coffman, 2006 The *Caenorhabditis elegans rhy-1* gene inhibits HIF-1 hypoxia-inducible factor activity in a negative feedback loop that does not include *vhl-1*. Genetics 174: 1205-1214.

Yang, Y., J. Lu, J. Rovnak, S. L. Quackenbush and E. A. Lundquist, 2006 SWAN-1, a Caenorhabditis elegans WD repeat protein of the AN11 family, is a negative regulator of Rac GTPase function. Genetics 174: 1917-1932.

Zhang, Y., Z. Shao, Z. Zhai, C. Shen and J. A. Powell-Coffman, 2009 The HIF-1 hypoxia-inducible factor modulates lifespan in *C. elegans*. PLoS One 4: e6348.
